# A method for forecasting the number of hospitalized and deceased based on the number of newly infected during a pandemic

**DOI:** 10.1038/s41598-022-08795-9

**Published:** 2022-03-21

**Authors:** Rudolf Scitovski, Kristian Sabo, Šime Ungar

**Affiliations:** 1 Department of Mathematics, University of Osijek, 31 000 Osijek, Croatia; 2 Department of Mathematics, University of Zagreb, 10 000 Zagreb, Croatia

**Keywords:** Viral infection, Health policy

## Abstract

In this paper we propose a phenomenological model for forecasting the numbers of deaths and of hospitalized persons in a pandemic wave, assuming that these numbers linearly depend, with certain delays $\tau >0$ for deaths and $\delta >0$ for hospitalized, on the number of new cases. We illustrate the application of our method using data from the third wave of the COVID-19 pandemic in Croatia, but the method can be applied to any new wave of the COVID-19 pandemic, as well as to any other possible pandemic. We also supply freely available *Mathematica* modules to implement the method.

## Introduction

In this paper we propose a phenomenological model for forecasting the number of deaths and of hospitalized persons in a pandemic wave, assuming that the number of deaths linearly depends, with a certain delay $\tau >0$, on the number of new cases, and similarly, that the number of hospitalizations linearly depends on the number of new cases with another delay $\delta >0$. The predictions are carried out every day using daily data on new cases, deaths and hospitalized from the beginning of the pandemic wave. The realization of this idea goes like this: first, for a certain day *T* during the pandemic wave, using the cumulative data on new cases up to that date, determine the growth model function of new cases $\Phi$. Then define the model function of deaths depending linearly on the function $t\mapsto \Phi '(t-\tau )$, where $\tau$ is the delay not known in advance. The parameters of the model function defined in this way are determined as the Least Squares (LS) problem for the data on deaths. The forecast for numbers of deaths can now be made for the next $\tau ^\star$ days starting from the day $T+1$, where $\tau ^\star$ is the optimal LS-value of the parameter $\tau$. In a similar way we define the model function for the hospitalized linearly depending on the function $t\mapsto \Phi '(t-\delta )$, where $\delta$ is the delay not known in advance. Repeating the procedure as for the deceased, the forecast for numbers of hospitalized is made for the next $\delta ^\star$ days.

Applying the proposed method to the third wave of the COVID-19 pandemic in Croatia showed that it was possible to make acceptable predictions of numbers of deaths for the next ten to twelve days, with the average death rate being about $1.8\%$. Also, it was possible to predict numbers of hospitalized in a period which includes the beginning of decrease of numbers of new cases, for the next 11 days, and earlier, during the progressive growth of numbers of new cases, for a considerably longer period.

When a pandemic brakes out it is a matter of utmost importance to estimate, as soon as possible, the progress of numbers of infected, deceased, hospitalized, and recovered cases. Since the COVID-19 pandemic broke forth, there has been an abundance of publications proposing, based on the available data, various mathematical models for short- and long-term forecasts, in order that the health authorities can plan and prepare resources to fight the pandemic.

Mathematical models for COVID-19 (and also for other epidemics or pandemics) may be classified as^1,2^: (1) transmission dynamics models, and (2) phenomenological models. The main purpose of transmission dynamics models^3–9^ is to describe the mechanisms underlying an epidemic and understand its behavior as well as the effects of human interventions. On the other hand phenomenological models^2,10,11^ do not aim at understanding the mechanisms underlying an epidemic, but can be used for efficient and rapid forecasts.

In the context of phenomenological models for of COVID-19 pandemics, proposed were: a mathematical model for estimating the case fatality risk, i.e. the proportion of deaths from a certain disease compared to the total number of people diagnosed with the disease for a particular period^12^; a mathematical model to determine the maximally allowed daily growth rates that lead away from exponential increase toward stable and declining numbers^13^; a method to predict the number of deaths^14^. In Ref.^15^ based on data for the first wave of the COVID-19 pandemic in the United States (US) and in the European Economic Area countries (EEA), a mathematical model using the Gauss model function was proposed for forecasting the necessary capacity of hospitals in the US and the EEA. In Ref.^16^ a framework was designed for assessing the predictive validity of COVID-19 mortality forecasts. The authors in Ref.^17^ study the factors associated with observed trends in the in-hospital mortality rates in the US during the first nine months of the COVID-19 pandemic.

Especially, often used in the literature was the logistic growth model and its generalizations^10,11,18–20^. For example, in Ref.^20^ it was analyzed the growth behavior of the top 25 most affected countries by means of a local slope analysis and three distinct patterns were identified which individual countries follow depending on the strictness of the lock-down protocols: rise and fall, power law, and logistic. Furthermore, in Ref.^11^ the authors proposed a new logistic model that is well-suited for power-law epidemic growth. It was shown that this model consistently makes accurate predictions of peak heights, peak locations and cumulative saturation values for incomplete epidemic growth curves.

Our paper is organized as follows. In the next section we propose a new method for forecasting, during a pandemic, the numbers of hospitalized and deceased based on the number of new cases. In “Application of the method to COVID-19 pandemic” we apply the proposed method to the third wave of COVID-19 pandemic in Croatia. Some conclusions are given in “Conclusions”, and in “Data and computer code availability” we describe our freely available software developed for the proposed method.

## Forecasting the numbers of deceased and hospitalized persons

Assume that in time of an epidemic we have at our disposal data $(t_i,n_i,d_i,h_i)$, $i=1,\dots ,m$, on the numbers of confirmed new cases $(n_i)$, newly deceased $(d_i)$, and the total numbers of hospitalized persons $(h_i)$ on day $t_{i}$, during a certain observed period $[t_1,t_m]$.

Hypothesizing that the number of deceased on the day *t* depends on the number of new cases at some previous day $t-\tau$, $\tau >0$, and that the number of newly hospitalized on the day *t* depends on the number of new cases on some previous day $t-\delta$, $\delta >0$, we will try to estimate the numbers of deceased in the upcoming period $[t_m,t_m+\tau ]$, and of hospitalized in the upcoming period $[t_m,t_m+\delta ]$, thus giving important indicators for monitoring and fighting the pandemic.

In order to operationalize these assumptions using the available data, we will first determine the corresponding growth model function $\Phi$ for the newly reported cases.

Due to different methodologies and dynamic of daily testing throughout the week, the daily data on new cases are not reliable (i.e. contain some random errors). Therefore, instead of using the daily data, we will estimate the values of parameters *a*, *b*, and *c* of the growth model function $\Phi$ based on the *cumulative* numbers $N_i=\sum \nolimits _{s=1}^i n_s$, $i=1,\dots ,m$, of new cases during the same period $[t_1,t_m]$. The derivative $t\mapsto \Phi '(t)$ will be used as the model function for the *daily* new cases in the period $[t_1,t_m]$.

For the growth model function $\Phi$ one can choose any of the well-known growth model functions: logistic or generalized logistic model function (see e.g. Refs.^21,22^), Gompertz model function (see e.g. Ref.^23^), or Weibul model function (see e.g. Ref.^24^). According to a large number of references on modeling the COVID-19 pandemic (see e.g. Refs.^11,19,20^), in this paper we will use the ordinary *Logistic model function*

1 $$\begin{aligned} \Phi (t):=y(t;a,b,c)=\frac{a}{1+b\,e^{-ct}},\quad a,b,c>0, \end{aligned}$$

which is the solution to the differential equation

2 $$\begin{aligned} y'=c\,y\,(a-y),\quad a,c>0, \end{aligned}$$

describing the following law: *The growth rate of the number of cases at the moment* *t is t* *proportional to the total number of cases at that moment, y*(*t*), *and the number of potentially new cases,* $(a-y(t))$.

The unknown parameters *a*, *b*, *c* will be determined by solving the nonlinear least squares problem^25,26^

3 $$\begin{aligned} \mathop {\mathrm {argmin}}\nolimits \limits _{a,b,c>0}\sum \limits _{i=1}^m (N_i-y(t_i;a,b,c))^2. \end{aligned}$$

The obtained parameters will be denoted $a^\star$, $b^\star$, and $c^\star$, and the model function $\Phi$ will be the function $t\mapsto y(t;a^\star ,b^\star ,c^\star )$.

The simple global optimization problems (GOP) (3) can be solved using the *Mathematica* module NonlinearModelFit^27^.

### Forecasting the number of deceased

As we already said, the dependence of the number of deceased on the number of new cases is based on the following assumptions:

(i) there is a time delay $\tau$ between infection and the death;
(ii) The number of deceased linearly depends on the derivative $\Phi '(t-\tau ;a^\star ,b^\star ,c^\star )$, where $a^\star$, $b^\star$, $c^\star$ are optimal parameters obtained by solving the nonlinear least squares problem (3) on the basis of cumulative data $(t_i,N_i)$, $i=1,\dots ,m$.

Knowing the model function $\Phi$ of the cumulative number of new cases, i.e. knowing the parameters $a^\star ,\,b^\star ,\,c^\star$, the function of daily deceased can be expressed by (see also similar models in Refs.^24,28,29^)

4 $$\begin{aligned} \varphi (t;u,\alpha ,\tau )=u+\alpha \, \Phi '(t-\tau ;a^\star ,b^\star ,c^\star ). \end{aligned}$$

The parameters *u*, $\alpha$ and $\tau$ of the model function $\varphi$ will be determined using data $(t_i,d_i)$, $i=\kappa ,\dots ,m$, on the numbers of deceased as the following GOP (data for the deceased are for $\kappa$, say 15, days later since we assume that the deaths before that belong to the previous pandemic wave):

5 $$\begin{aligned} \smash [t]{\mathop {\mathrm {argmin}}\nolimits \limits _{u,\alpha ,\tau >0}F(u,\alpha ,\tau ),\qquad F(u,\alpha ,\tau )=\sum \limits _{i=\kappa }^m (d_i-\varphi (t_i;u,\alpha ,\tau ))^2}. \end{aligned}$$

The solution to this problem will be denoted $(u^\star , \tau ^\star , \alpha ^\star )$: $\tau ^\star$ is the average period (in days) between new cases and the corresponding deaths, and $\alpha ^\star$ represents the proportion of deceased among the new cases reported $\tau ^\star$ days earlier.

Since in general, the number $\tau ^\star$ won’t be an integer, we will denote by $\hat{\tau }=\lfloor \tau ^\star \rceil$ the nearest integer. Therefore, the number of deceased during the period $[t_{m+1},t_m+\hat{\tau }\:]$ of $\hat{\tau }$ days can be calculated using the formula

6 $$\begin{aligned} \sum \limits _{t=t_m+1}^{t_m+\hat{\tau }} \lfloor \varphi (t;u^\star ,\alpha ^\star ,\tau ^\star )\rceil . \end{aligned}$$

This procedure should be repeated, by adding new data, each day.

#### Remark 1

Because of numerical sensitivity of the GOP (5), we will solve this problem similarly as was done in Refs.^30,31^: first we determine the initial approximation using the global optimization algorithm DIRECT^32–34^, and then we apply the Newton optimization method^25,26^ by using *Mathematica* module NonlinearModelFit. The same will be done to solve the GOP (9).

### Forecasting the number of hospitalized

The connection between the numbers of hospitalized and of newly infected is more complicated than in the case of deceased. Namely, the total number $h_i$ of hospitalized on day $t_i$ can be defined as

7 $$\begin{aligned} h_i=h_{i-1}+\nu _i-\rho _i-d_i, \end{aligned}$$

where $\nu _i$ is the number of those admitted to hospital on day $t_i$, $\rho _i$ is the number of released from hospital on day $t_i$, and $d_i$ is the number of patients who died on day $t_i$. Note that $\nu _i-\rho _i-d_i$ denotes the change (the growth or decrease) of the number of hospitalized patients on day $t_i$. We will assume the following:

(i) there is a time delay $\delta$ between new cases and new hospitalizations;
(ii) The number of hospitalized linearly depends on the derivative $\Phi '(t-\delta ;a^\star ,b^\star ,c^\star )$, where $a^\star$, $b^\star$, $c^\star$ are optimal parameters obtained by solving the nonlinear least squares problem (3) on the basis of cumulative data $(t_i,N_i)$, $i=1,\dots ,m$.

Based on these assumptions and knowing the growth model function $t\mapsto \Phi (t;a^\star ,b^\star ,c^\star )$ for the cumulative number of new cases, the estimated number of hospitalized can be expressed as (see also similar models in Refs.^28,29^)

8 $$\begin{aligned} \psi (t;u,v,\delta )=u+ v\,\Phi '(t-\delta ;a^\star ,b^\star ,c^\star ), \end{aligned}$$

where the parameters *u*, *v*, and $\delta$ of the model function $\psi$ will be determined using data $(t_i,h_i)$, $i=\kappa ,\dots ,m$, on the numbers of hospitalized persons, as the following GOP:

9 $$\begin{aligned} \mathop {\mathrm {argmin}}\nolimits \limits _{u,v,\delta >0}F(u,v,\delta ),\qquad F(u,v,\delta )=\sum \limits _{i=\kappa }^m (h_i-\psi (t_i;u,v,\delta ))^2, \end{aligned}$$

where data for the hospitalized persons are for $\kappa$, say 15, days later since we assume that hospitalizations before that belong to the previous wave. The solution to this problem will be denoted $(u^\star ,v^\star ,\delta ^\star )$. The number $\delta ^\star$ is the average period (in days) between new cases and the corresponding hospitalizations.

The number $h_i$ of hospitalized on the day $t_i$, $i>m$, can now be estimated as

10 $$\begin{aligned} h_i\approx u^\star +v^\star \,\Phi '(t_i-\delta ^\star ;a^\star ,b^\star ,c^\star ). \end{aligned}$$

Comparing (7) and (10), we have

11 $$\begin{aligned} \nu _i-\rho _i-d_i \approx v^\star \Phi '(t_i-\delta ^\star )-v^\star \Phi '(t_{i-1}-\delta ^\star ) \approx v^\star \,\Phi ''(t_i-\delta ^\star )= \psi '(t_i,u^\star ,v^\star ,\delta ^\star ). \end{aligned}$$

Therefore, the change (the growth or decrease) $\nu _i-\rho _i-d_i$ of the number of patients hospitalized on day $t_i$, $i>m$, can be expressed as the value $\psi '(t_i,u^\star ,v^\star ,\delta ^\star )$.

Important points of the function $\psi '$ are its maximum *M* which is reached at

12 $$\begin{aligned} t_M=\delta ^\star +\tfrac{\ln (b^\star (2-\sqrt{3}))}{c^\star }, \end{aligned}$$

and its null point $T_0=(t_0,0)$. One can say that the number of hospitalized has a *progressive growth* until the moment $t_M$, and a *degressive growth* after that, and that the number of hospitalized decreases after the moment $t_0$.

Since in general, the number $\delta ^\star$ is not an integer, we will denote by $\hat{\delta }=\lfloor \delta ^\star \rceil$ the nearest integer. Therefore, the predicted numbers of hospitalized during the next period of $\hat{\delta }$ days are

13 $$\begin{aligned} \lfloor \psi (t_m+1;u^\star ,v^\star ,\delta ^\star )\rceil ,\;\dots ,\;\lfloor \psi (t_m+\hat{\delta };u^\star ,v^\star ,\delta ^\star )\rceil . \end{aligned}$$

This procedure should be repeated, by adding new data, each day.

We also developed software support for the proposed method in form of *Mathematica* modules, see “Data and computer code availability”.

## Application of the method to COVID-19 pandemic

We will test our proposed method on data for the third wave of COVID-19 pandemic in Croatia, predicting the numbers of hospitalized and of deceased persons.

### COVID-19 pandemic in Croatia

**Figure 1:**
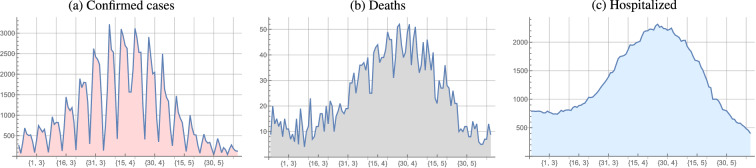
Daily numbers of new cases, deaths and hospitalized since February 20, 2021.

We will use the daily data on new cases (Fig. 1a), deaths (Fig. 1b), and hospitalized (Fig. 1c) in Croatia, available at https://covid.ourworldindata.org/data/owid-covid-data.xlsx, starting with February 20, 2021 when the third wave of the pandemic in Croatia was declared. This will be the first day, numbered $t_1=1$. June 7 will be considered the last day of that pandemic wave.

Since we assumed that the numbers of deceased and of hospitalized lag behind the number of new cases by several days, we will consider data on new cases starting from February 20, 2021, and data on deceased and hospitalized starting from March 8, 2021, i.e. 15 days later, presuming that the deaths and hospitalizations between these dates were still related to the previous wave of the pandemic.

Our model is conceived so that we look at the current data every day, and according to these data estimate the probable future numbers of deaths and hospitalized.

Let $(t_i,n_i,d_i,h_i)$, $i=1,\dots ,m$, be the pandemic data available on the day $t_m$. In order to test our method, we divide this data into two parts: the *training data* $(t_i,n_i,d_i,h_i)$, $i=1,\dots ,T$, and the *testing data* $(t_i,n_i,d_i,h_i)$, $i=T+1,\dots ,m$. The training data will be used to estimate the optimal parameters, and the testing data to evaluate the accuracy of our method.

#### Forecasting the number of deceased

Using our data, it turned out that the estimated numbers of deceased in the period $[T,T+\hat{\tau}\:]$ closely followed the numbers of actual deaths for almost every $T>50$. The relevant calculations using the supplied software (see “Data and computer code availability”) were carried out for every $T\in [50,75]$ (April 10 through May 5). The average delay $\hat{\tau }$ for such *T*’s was 12.84 with standard deviation 1.77, and the proportion of the deceased among new cases $\hat{\tau }$ days earlier was $1.74\%$ on average, with standard deviation 0.08.

**Figure 2:**
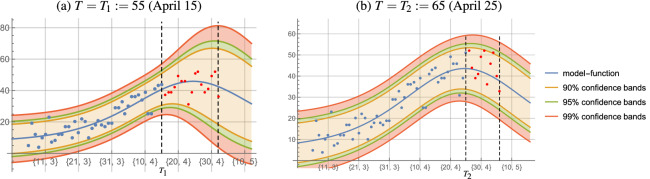
Graphs of the model function $\varphi$ and confidence bands for the fit.

As an illustration we first consider training data in the period of progressive growth of new cases with $T=T_1:= 55$, i.e. April 15 (see the first dashed vertical line in Fig. 2a).

The optimal parameters $(a^\star _1, b^\star _1, c^\star _1)$ of the logistic model function will be determined using the training data $(t_i,N_i)$, $i=1,\dots ,T_1$, of the cumulative numbers of new cases, while the optimal parameters $u^\star _1$, $\alpha ^\star _1$ and $\tau ^\star _1$ defining the function $\varphi$ given by (4), will be determined using the training data $(t_i,d_i)$, $i=\kappa ,\dots ,T_1$, for the deceased during the same period, by solving the GOP (5). The obtained results are shown in Table 1.

Using the testing data for the deceased, the same table shows the number of actual deaths during $\hat{\tau }_1=\lfloor \tau ^\star _1\rceil =17$ days starting on April 16, and the number predicted by the formula (6). Note that for this 17-day period the prediction differs little from the actual number of deceased. Furthermore, the proportion of deceased among new cases from 17 days earlier is $\alpha _1^\star \approx 2\%$ (see Table 1).

**Table 1:** Optimal parameters for the logistic growth model function $\Phi$ and for the model function $\varphi$ of delayed deaths (4), using training data $(t_i,n_i,d_i)$, $i=1,\dots ,T$ with $T=T_1:=55$ and $T=T_2:=65$, and the comparison between the numbers of predicted and of actual deaths in the period $[T,T+\hat{\tau}\:]$.

T	Parameters	Parameters	Delay	$[T,T+\hat{\tau}\:]$		
	$(a^\star , b^\star , c^\star )$	$(u^\star , \alpha ^\star , \tau ^\star )$	$\hat{\tau }$	Predicted	Actual	Error
$T_1:=55$	(95961.3, 47.03, 0.081)	(7.317, 0.020, 16.93)	17	756	732	$3.3 \%$
$T_2:=65$	(121443.0, 50.22, 0.073)	(5.681, 0.017, 11.16)	11	453	483	$6.2 \%$

Figure 2a shows the graph of the model function $\varphi$ given by (4) (blue curve), where the function $\Phi$ was determined beforehand as the logistic model function for the cumulative number of new cases. The red dots represent the actual daily numbers of deaths during the first 17 days after April 15 (732 deaths in total). Visualized are also the $90\%$, $95\%$, and $99\%$ confidence bands for the fit of the function $\varphi$, justifying the obtained results. The vertical dashed lines bound the range of data between *T* and $[T+\hat{\tau }\:]$.

In a similar way we will analyze the training data in a period which includes the beginning of decrease of numbers of new cases with $T=T_2:=65$, i.e. April 25 (see the first dashed vertical line in Fig. 2b). The optimal parameters $(a^\star _2, b^\star _2, c^\star _2)$ of the logistic model functions in this case, as well as the parameters $u^\star _2, \alpha ^\star _2$, $\tau ^\star _2$ of the corresponding function $\varphi$ are also shown in Table 1.

Using the testing data for the deceased, the same table shows the number of actual deaths during $\hat{\tau }_2=\lfloor \tau ^\star _2\rceil =11$ days starting on April 26, and the number predicted by the formula (6). Furthermore, the proportion of deceased among new cases from eleven days earlier is $\alpha _2^\star \approx 1.7\%$.

Figure 2b shows the graph of the model function $\varphi$, where the function $\Phi$ was determined as before. The red dots represent the actual daily numbers of deaths during the first eleven days after April 25 (483 deaths in total). Visualized are also the $90\%$, $95\%$, and $99\%$ confidence bands for the fit of the function $\varphi$, justifying the obtained results.

#### Forecasting the number of hospitalized persons

The parameters of the model function (8) will be determined based on the training data $(t_i,N_i)$, $i=1,\dots T$, of cumulative numbers of new cases. After that, the optimal parameters $u^\star$, $v^\star$, and $\delta ^\star$ of the model function $\psi$ which, according to formula (8), relate the number of hospitalized to the number of new cases, will be estimated by solving the GOP (9) based on the training data $(t_i,h_i)$, $i=\kappa ,\dots ,T$, of hospitalized persons. This GOP will again be solved as was described in Remark 1. In this way one obtains numbers

$$\begin{aligned} \lfloor \psi (t_{T+1};u^\star ,v^\star ,\delta ^\star )\rceil ,\;\dots ,\;\lfloor \psi (t_{T+\hat{\delta }};u^\star ,v^\star ,\delta ^\star )\rceil , \end{aligned}$$

predicting the numbers of hospitalized for the next $\hat{\delta }$ days starting with day $t_{T+1}$. These numbers have to be compared with numbers of actually hospitalized during the same period. This comparison can be carried out using various measures, see e.g. Ref.^35^, and we will use the *Mean Absolute Percentage Error* (MAPE).

Applying the aforementioned software, described later in “Data and computer code availability”, to our data for $T\in [45,75]$ (April 5 through May 5), shows that for $T\ge 48$ the $\texttt {MAPE}\le 10\%$, inferring that the forecast is acceptable. Delays drop from 21, and after $T=62$ stabilize at 11.

As an illustration, similarly as in “Forecasting the number of deceased”, we first consider training data in the period of progressive growth of new cases with $T=T_1:=50$ (April 10), and then the training data in a period which includes the beginning of decrease of numbers of new cases with $T=T_2:=70$ (April 30).

**Figure 3:**
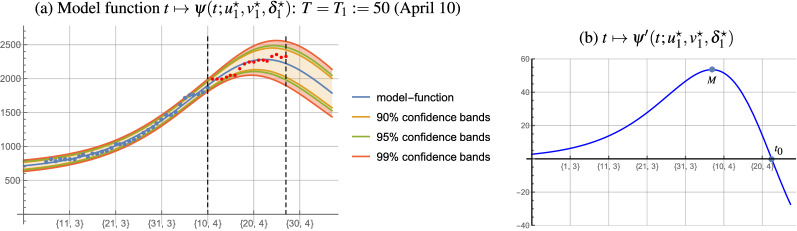
Numbers of hospitalized, the model function $\psi$ with confidence bands for the fit, and the derivative $\psi '$ ($T_1:=50$).

The optimal parameters of the logistic model function for the cumulative number of new cases for $T=T_1:=50$ are $a^\star =86231.7$, $b^\star =45.49$, and $c^\star = 0.085$. Solving the GOP (9) for $T=T_1:=50$ we obtain the optimal parameters $u^\star _1=638.94$, $v^\star _1=0.900$, and $\delta ^\star _1=17.35$ of the model function $\psi$ given by (8), and therefore the average delay equals $\hat{\delta }_1=17$. Figure 3a shows the graph of the model function $\psi$ (blue curve). Red dots represent the actual numbers of hospitalized during the 17-day period following April 10. Visualized are also $90\%$, $95\%$, and $99\%$ confidence bands for the fit of the function $\psi$, justifying the obtained results. The measure $\texttt {MAPE}=3\%$ infers a high degree of agreement between our forecast and the actual number of hospitalized during the 17-day period from April 11 to April 28, which is also visible in Fig. 3a. The maximum of the derivative $\psi '$ is, according to (12), reached on April 7, and its null point is on April 22 (see Fig. 3b). This means that until April 7 the growth of the number hospitalized patients is progressive, then, until April 22, digressive, and after April 22 the number of hospitalized drops.

**Figure 4:**
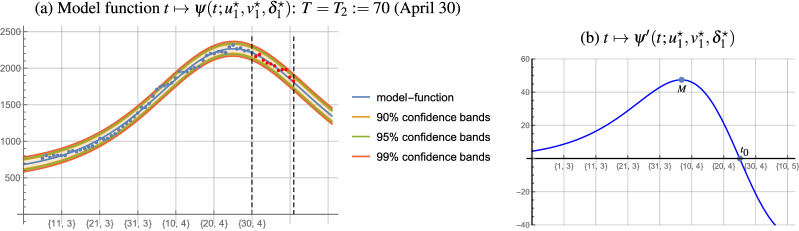
Numbers of hospitalized, the model function $\psi$ with confidence bands for the fit, and the derivative $\psi '$ ($T_2:=70$).

The optimal parameters of the logistic model function for the cumulative number of new cases for $T=T_2:=70$ (April 30) are $a^\star =125072.0$, $b^\star =50.16$, and $c^\star = 0.072$. Solving the GOP (9) for $T=T_2:=70$ we obtain the optimal parameters $u^\star _2=557.34$, $v^\star _2=0.758$, $\delta ^\star _2=10.71$, and the average delay is $\hat{\delta }_2=11$. The measure $\texttt {MAPE}=3.0\%$ infers a high degree of agreement between our forecast and the actual numbers during the 11-day period starting on April 30, which is also visible in Fig. 4a. The maximum of the derivative $\psi '$ is reached on April 7, and its null point is on April 25 (see Fig. 4b), meaning that until April 7 the growth of the number hospitalized is progressive, then until April 25 digressive, and after April 25 the number of hospitalized drops.

## Conclusions

In our paper we propose a phenomenological model for forecasting the number of deaths and hospitalizations in a pandemic wave, assuming that the number of deaths linearly depends, with a certain delay $\tau >0$, on the number of new cases, and similarly, that the number of hospitalizations linearly depends on the number of new cases with another delay $\delta >0$. The predictions should be carried out every day using daily data on new cases, deaths and hospitalized from the beginning of the pandemic wave. For this purpose we devised dedicated *Mathematica* notebooks, freely available at http://models.mathos.unios.hr/.

The method is tested and illustrated using data on the third wave of the COVID-19 pandemic in Croatia, but the method can be applied to any new wave of the COVID-19 pandemic, as well as to any other possible pandemic.

In the meantime, since the first version of this paper was written, numerous results appeared where various authors dealt with the same problem differently (see e.g. Refs.^1,11,18–20^). The basic strength of our approach is its simplicity based on our previous work^21–23,29^, and using efficient numerical methods for finding optimal parameters of the model (see Remark 1), which is also based on our previous work. Besides daily data on deaths and hospitalized, our model uses data on confirmed infected cases. But provided that a good method for detecting or some other way for coming up with *actual* numbers of infected persons were found, the data on confirmed cases in our model could easily be replaced by the data on actually infected, making the predictions equally simple and fast, but considerably more accurate and reliable. Our model could be enhanced by using also the vaccination data.

## Data and computer code availability

All evaluations were done using our own *Mathematica* modules Dead[] and Hospitalized[], freely available at http://models.mathos.unios.hr/. Both modules call the data set Data.txt, also available at this url, containing the numbers of new cases, deceased, and hospitalized during the third wave of the COVID-19 pandemic in Croatia.

The module Dead[] outputs the forecast for cumulative numbers of deceased for the next $\hat{\tau }$ days and the proportion of deceased among cases reported $\hat{\tau }$ days earlier (see “Forecasting the number of deceased”). The results are given for the logistic and Gompertz model functions. The module can also output the following:

- the bar charts of the new cases and of the deceased;
- optimal parameters of the corresponding logistic and Gompertz model functions of cumulative numbers of new cases, their graphs, characteristic points and saturation levels, see Refs.^22,23^;
- optimal parameters of the relating function $\varphi$ given by (4), its graph, confidence bands for the fit, characteristic points and the proportion of the deceased using the logistic and Gompertz model functions;

The module Hospitalized[] outputs the forecast for numbers of hospitalized during the next $\hat{\delta }$ days (see “Forecasting the number of hospitalized”). The results are given using the logistic model function. Additionally, the module can also output the following:

- the bar charts of the new cases and of the hospitalized;
- optimal parameters of the logistic model function of cumulative numbers of new cases, its graph, and the characteristic points, see Ref.^22^;
- optimal parameters $u^\star$, $v^\star$, and $\delta ^\star$ of the relating function $\psi$ given by (8), its graph, confidence bands for the fit, and the characteristic points;
- the value of MAPE.
